# Association of TNF-α genetic polymorphisms with recurrent pregnancy loss risk: a systematic review and meta-analysis

**DOI:** 10.1186/s12958-016-0140-6

**Published:** 2016-02-02

**Authors:** Hui-Hui Li, Xing-Hua Xu, Jing Tong, Kai-Yue Zhang, Cong Zhang, Zi-Jiang Chen

**Affiliations:** Center for Reproductive Medicine, Ren Ji Hospital, School of Medicine, Shanghai Jiao Tong University, Shanghai, 200135 China; Shanghai Key Laboratory for Assisted Reproduction and Reproductive Genetics, Shanghai, 200135 China; Center for Reproductive Medicine, Shandong Provincial Hospital Affiliated to Shandong University; Shandong Provincial Key Laboratory of Reproductive Medicine, Jinan, 250021 China; National Research Center for Assisted Reproductive Technology and Reproductive Genetics, The Key laboratory for Reproductive Endocrinology of Ministry of Education, Jinan, 250021 China; Department of Gynecology and Obstetrics, Liaocheng People’s Hospital, Liaocheng, 252000 China

**Keywords:** Meta-analysis, polymorphism, recurrent pregnancy loss (RPL), tumor necrosis factor alpha (TNF-α)

## Abstract

**Background:**

Several studies on the association of tumor necrosis factor alpha (TNF-α) polymorphisms with recurrent pregnancy loss (RPL) risk have reported conflicting results. The present meta-analysis was conducted to provide a more precise estimation of these relationships and to investigate the real association between TNF-α polymorphisms and RPL.

**Methods:**

An extensive eligible literature search for relevant studies was conducted on PubMed, Embase, and The Cochrane Library from their inceptions to May 12, 2015. Specific inclusion criteria were used to evaluate articles. The odds ratio (OR) with 95% confidence intervals (CIs) were used to assess the strength of associations. Statistical analyses were performed by the STATA12.0 software.

**Results:**

10 case–control studies including 1430 RPL patients and 1727 healthy controls were identified. Meta-analysis indicated that TNF-α-308G/A (rs1800629) polymorphism in the TNF-α gene correlated with elevated RPL risk whereas no significant association was observed between TNF-α-238G/A (rs361625) and RPL.

**Conclusions:**

The current meta-analysis demonstrates that TNF-α-308G/A polymorphism in the TNF-α gene is associated with susceptibility to RPL.

## Background

Recurrent pregnancy loss (RPL) is defined as three or more consecutive spontaneous abortions before the 20th week of gestation [1, 2]. It is estimated that RPL affects approximately 3% of healthy women of reproductive age with undetermined causes [2, 3]. Until now, a few known etiological factors have been considered as the cause of RPL including genetic defects such as parental chromosome abnormalities, endocrine and metabolic disorders such as hypothyroidism, luteal phase deficiency and diabetes mellitus, autoimmune abnormalities such as antiphospholipid syndrome [4–6], although the mechanisms are largely unknown.

Some studies have led to the awareness that these unexplained RPL might be due to dysregulated immunologic factors [7, 8]. Considerable evidence has accumulated indicating that cytokines play a major role in reproductive events [9]. For instance, tumor necrosis factor-α (TNF-α) is a potent cytokine which produced by mononuclear phagocytes, natural killer (NK) cells, and antigen-stimulated T-cells [10]. It has often been associated with increased risk for adverse pregnancy outcomes. Circulating levels of TNF-α are higher both in animals and humans with a miscarriage compared to those with a successful pregnancy, suggesting that this cytokine is exclusively harmful for pregnancy [8, 11–13].

An increasing number of genetic association research are conducted to determine the genetic background of RPL [14]. Research efforts have focused on single nucleotide polymorphisms (SNP) because cytokines have their important roles in implantation and gestation [14]. The production of cytokines can be controlled by genetic polymorphisms, especially in the promoter regions. The TNF-α is located within the human leukocyte antigen class III region in chromosome 6p21.3and has several functional sites of polymorphisms [15]. Variants in the TNF-α promoter region were previously implicated in the pathogenesis of RPL, hence, many studies have been directed towards the relationships between SNPs in the promoter region of TNF-α at -1031T/C, −863C/A, −857C/T, −376G/A, −308G/A, −238G/A, +488G/A and RPL [16, 17]. Although many studies have associated RPL and TNF-α polymorphisms, their role in reproductive failure is still debated. Some studies demonstrated that the −308 G/A polymorphism is not associated with RPL [18, 19], other studies gave significant evidence for an increased risk of RPL for the carriers of the TNF-α-308A allele [17, 20].

As stated above, several original studies have reported the correlations between TNF-α polymorphism and RPL, but the results are unconvincing and unreliable, which may partly be due to the relatively small samples and different human populations. A previous meta-analysis was conducted in 2012 trying to investigate this relationship [21]. In view of 12 eligible studies, the results indicate that TNF-α-308G/A, −238G/A polymorphisms are not significantly associated with the risk of RPL in the overall population. However, careful inspection of the data used in that study revealed a noteworthy inconsistency of diagnostic criteria and much stricter entry criteria was needed to clarify such inconsistencies that might confound the conclusions [22]. In the past three years, several more replication researches performed to reevaluate the effect of TNF-α gene polymorphisms on RPL provided some new data and diverse conclusions [17–20]. Accordingly, we performed a meta-analysis with much stricter entry criteria to investigate the association between TNF-α polymorphism and RPL risk.

## Methods

### Search strategy

Article searches were performed independently by two investigators and the final search strategies were performed with agreement. An extensive systematic literature search for relevant studies was conducted with PubMed, Embase, and The Cochrane Library from their earliest available date through May 12, 2015. For TNF-α polymorphisms and RPL risk, the search terms were as follows: (“tumor necrosis factor” OR “TNF”) AND (“recurrent pregnancy loss” OR “recurrent spontaneous miscarriage” OR “recurrent spontaneous abortion”) AND “polymorphism”. All the articles about three or more miscarriages associated with TNF-α polymorphism were included. Moreover, all articles were published in the primary literature to avoid duplicating analyses. All clearly irrelevant studies, editorials, case reports, and review articles were excluded. Furthermore, literatures cited in the reference sections of review articles and other relevant studies were searched manually for additional eligible studies.

### Selection criteria

Eligible studies were selected according to the following explicit inclusion criteria:

(1) the original study was designed as an independent genotyped case–control study; (2) inclusion of both RPL cases and non-RPL controls; (3) investigation of the correlation between TNF-α genetic polymorphisms and RPL risk; (4) adequate data that could be used to calculate the numbers of genotype frequency had to be clearly described in the original study. In addition, the following exclusion criteria were also used: (1) no healthy control population and raw data; (2) genotype frequency unavailable; (3) non-conformity with the criteria for RPL; and (4) duplication of previous publications.

### Data extraction

The bibliographic search and data extraction were conducted independently by two investigators from all eligible publications according to the above inclusion criteria. Any disagreement was subsequently resolved by consensus with a third author. The following characteristics was collected prospectively: the first author’s name, year of publication, source of publication, country of origin, genotype number in cases and controls, genotype method, and gene polymorphism (Table 1).

**Table 1:** Main characteristics of the studies included in the meta-analysis

Gene polymorphism	Author	Year	Country	Diagnostic Criteria(numbers of consecutive pregnancy losses)	Genotype		Genotype method	Quality Assessment
					Case	Control		
-238G/A	Alkhuriji A.F	2013	Saudi	three or more	57/8/0^a^	55/7/3^a^	PCR	1: adequate; 2: not stated;3: adequate;
								4: adequate; 5: unequal
	Gupta R.	2012	Indian	three or more	121/63/16^a^	154/113/33^a^	PCR–RFLP	1: adequate; 2: not stated;3: adequate;
								4: adequate; 5: not stated
	Finana R.R.	2010	Bahrain	three or more	148/52/4^a^	200/48/0^a^	PCR–RFLP	1: adequate; 2: not stated;3: adequate;
								4: adequate; 5: unequal
	Zammiti W	2009	Tunisia	three or more	264/88/20^a^	215/52/7^a^	PCR–RFLP	1: adequate; 2: not stated;3: adequate;
								4: adequate; 5: unequal
-308G/A	Alkhuriji A.F	2013	Saudi	three or more	33/24/8^a^	47/14/4^a^	PCR	1: adequate; 2: not stated;3: adequate;
								4: adequate; 5: unequal
	Gupta R.	2012	Indian	three or more	229/62/9^a^	425/70/5^a^	PCR–RFLP	1: adequate; 2: not stated;3: adequate;
								4: adequate; 5: unequal
	Kuar A.	2011	Indian	three or more	39/6/5^a^	41/7/2^a^	PCR–RFLP	1: adequate; 2: not stated;3: adequate;
								4: adequate; 5: unequal
	Finana R.R.	2010	Bahrain	three or more	164/32/8^a^	212/32/4^a^	PCR–RFLP	1: adequate; 2: not stated;3: adequate;
								4: adequate; 5: not stated
	Zammiti W	2009	Tunisia	three or more	319/39/14^a^	222/47/5^a^	PCR–RFLP	1: adequate; 2: not stated;3: adequate;
								4: adequate; 5: not stated
	Kamali- Sarvestani E	2005	Iranian	three or more	117/14^b^	122/21^b^	PCR	1: adequate; 2: not stated;3: adequate;
								4: adequate; 5: not stated
	Prigoshin N	2004	Argentina	three or more	35/6^b^	49/5^b^	PCR-SSP	1: adequate; 2: not stated;3: adequate;
								4: adequate; 5: unequal
	Pietrowski D	2004	Germany	three or more	133/33/2^a^	167/41/4^a^	PCR	1: adequate; 2: not stated;3: adequate;
								4: adequate; 5: not stated
	Daher S	2003	Brazil	three or more	36/12^b^	89/19^b^	PCR	1: adequate; 2: not stated;3: adequate;
								4: adequate; 5: unequal
	Babbage S.J.	2001	UK	three or more	30/13^b^	56/17^b^	PCR	1: adequate; 2: not stated;3: adequate;
								4: adequate; 5: unequal

Note: ^a^Genotype, for TNF-308G/A, GG/GA/AA; for TNF-238G/A, GG/GA/AA. ^b^Genotype, for TNF-308G/A, GG/GA+AA; for TNF-238G/A, GG/GA+AA

PCR, polymerase chain reaction; RFLP, restriction fragment length polymorphism

### Quality assessment

The quality of the included studies was assessed according to the following criteria from the previous report [23].

1. Description of the case and control subjects’ characteristics (adequate, inadequate);
2. Assessment and validation of miscarriage in the patients (adequate, inadequate, not stated). Adequate validation would include confirmation by scan or pathological examination; inadequate validation would include recollection of the patient as the only evidence or a biochemical pregnancy without ultrasound evidence of pregnancy;
3. Description of the laboratory procedures for the genotyping (adequate, inadequate);
4. Elimination of confounding factors in patients (not described, inadequate, adequate). “Adequate” refers to the elimination of the proven causes of recurrent miscarriage (chromosomal abnormalities of the couples, antiphospholipid antibodies, uterine abnormalities, protein C/S/antithrombin-III deficiency);
5. Equal assessment for confounding factors in the case and control groups (equal, unequal, not stated).

### Statistical analysis

Data management and analysis were performed using the programs STATA version 12 (StataCorp LP, College Station, TX, USA). Crude odds ratios (ORs) with 95% confidence intervals (CIs) were used to assess the association between the TNF-α polymorphisms and the risk of RPL. Analysis of polymorphisms was conducted in at least three studies. The ORs was calculated for the allele model, homozygote comparison, heterozygote comparison, dominant model, and recessive model based on the genotype frequencies in cases and controls. Heterogeneity was evaluated with Cochran's Q test and I^2^ statistic. When *P* value of Q test was less than 0.05 and/or I^2^ more than 50%, it was considered there was significant heterogeneity and a random-effects model was used, otherwise, the fixed effects model was selected. Potential publication bias was diagnosed statistically via the funnel plots and Egger’s tests. Moreover, subgroup analyses were conducted to explore reasons for heterogeneity. In all analyses, two-sided *P* value <0.05 was considered to be statistically significant.

## Results

### Study characteristics

**Fig. 1:**
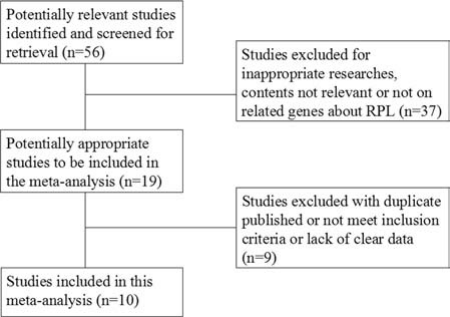
The process flow diagram of selected articles on genetic studies of TNF-α polymorphisms with RPL

We identified 19 articles that evaluated the association between TNF-α gene SNPs and RPL risk. 10 of these studies met the eligibility criteria defined in the Materials and Methods section [16–20, 24–28]. The main reasons for exclusion were as follows: 1 was duplicate publication [29], 3 were lack of clear data [30–32], and the other 5 excluded studies diagnosed RPL with at least two consecutive spontaneous abortions [33–37]. The detailed steps of our literature search are shown in Fig. 1.

The 10 studies reported here were published between 2001 and 2013 and involved totals of 1430 cases and 1727 controls. Overall, 5 studies were conducted in Asia, 2 in South America, 2 in Europe, and 1 in Africa. Among all the SNPs of the TNF-α gene addressed, −308G/A, and-238G/A were the most common. The DNA samples were extracted from blood in the included studies. Methods used for genotyping include direct DNA sequencing and polymerase chain reaction-restriction fragment length polymorphism (PCR-RELP). The main characteristics of all the included studies are summarized in Table 1.

### Meta-analysis results

#### −308 G/A and RPL Risk

**Fig. 2:**
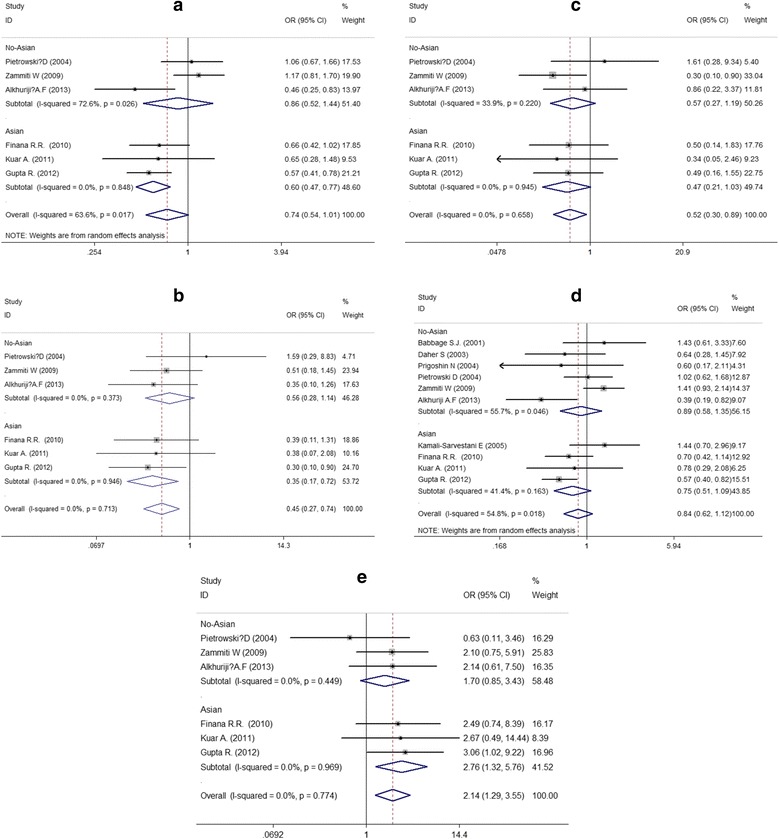
Forest plots for the associations between -308G/A polymorphism and RPL risk (**a** codominant genetic models; **b** homozygous genetic models; **c** heterozygous genetic models; **d** dominant genetic models; **e** recessive genetic models)

The combined results of all analyses indicated that the -308G/A polymorphism increased the risk of RPL in the homozygous comparison (AA vs. GG: OR = 0.445, 95% CI 0.268-0.741, *P* = 0.002) (Fig. 2b), the heterozygous model (AA vs. GA: OR = 0.519, 95% CI 0.303-0.89, *P* = 0.017) (Fig. 2c) and the recessive model (AA vs. GA + GG: OR = 2.141, 95% CI 1.291-3.55, *P* = 0.003) (Fig. 2e), but no significant associations were found in the codominant and dominant models. The results were as follows: A vs. G (OR = 0.737, 95% CI 0.539-1.01, *P* = 0.057) (Fig. 2a), AA + GA vs. GG (OR = 0.836, 95% CI 0.622-1.124, *P* = 0.236) (Table 2, Fig. 2d).

**Table 2:** Meta-analysis results for the two studied polymorphisms and RPL risk

Gene polymorphism	Inherited model	Heterogeneity-test		Analysis model^a^	Pooled OR (95% CI)	*P*
		*P* for Q test	*I* ^*2*^ (%)			
-238G/A	Codominant (A vs. G)	0.001	80.7	REM	0.996 [0.932,1.065]	0.912
	Homozygous (AA vs. GG)	0.017	70.7	REM	0.830 [0.238, 2.887]	0.769
	Heterozygous (AA vs. GA)	0.175	39.5	FEM	0.888 [0.54, 1.46]	0.640
	Dominant(AA+GA vs. GG)	0.007	75.2	REM	0.908 [ 0.566, 1.456]	0.690
	Recessive(AA vs. GA+GG)	0.036	64.9	REM	1.212 [ 0.394, 3.729]	0.737
-308G/A	Codominant (A vs. G)	0.017	63.6	REM	0.737 [0.539, 1.01]	0.057
	Homozygous (AA vs. GG)	0.713	0	FEM	0.445 [0.268, 0.741]	0.002
	Heterozygous (AA vs. GA)	0.658	0	FEM	0.519 [0.303, 0.89]	0.017
	Dominant(AA+GA vs. GG)	0.018	54.8	REM	0.836 [0.622, 1.124]	0.236
	Recessive(AA vs. GA+GG)	0.774	0	FEM	2.141 [1.291, 3.55]	0.003

Stratification by geographic position showed that the polymorphism of -308G/A was significantly associated with RPL for Asians rather than non-Asians under codominant, homozygous and recessive genetic models. The pooled ORs were 0.601(95%CI 0.468-0.773, *P* < 0.001), 0.345 (95% CI 0.165-0.723, *P* = 0.005), and 2.759 (95% CI 1.322-5.760, *P* = 0.007) for Asians under codominant, homozygous and recessive genetic models respectively. The results of this subgroup analysis are displayed (Table 3, Fig. 2).

**Table 3:** Results of subgroup analysis

Gene polymorphism	Inherited model	Subgroup	Heterogeneity -test		Analysis model^a^	Pooled OR (95% CI)	*P*
			*P* for Q test	*I* ^*2*^ (%)			
-308G/A	Codominant (A vs. G)	Asian	0.848	0	REM	0.601 [0.468, 0.773]	<0.001
		No-Asian	0.026	72.6	REM	0.864 [0.518, 1.443]	0.577
	Homozygous (AA vs. GG)	Asian	0.946	0	FEM	0.345 [0.165, 0.723]	0.005
		No-Asian	0.373	0	FEM	0.561 [0.277, 1.136]	0.109
	Heterozygous (AA vs. GA)	Asian	0.945	0	FEM	0.467 [0.213, 1.026]	0.058
		No-Asian	0.220	33.9	FEM	0.569 [0.272, 1.192]	0.135
	Dominant (AA+ GA vs. GG)	Asian	0.163	41.4	REM	0.747 [0.513, 1.089]	0.130
		No-Asian	0.046	55.7	REM	0.886 [0.581, 1.351]	0.573
	Recessive (AA vs. GA+GG)	Asian	0.969	0	FEM	2.759 [1.322, 5.76]	0.007
		No-Asian	0.449	0	FEM	1.703 [0.846, 3.426]	0.136

#### -238G/A and RPL Risk

**Fig. 3:**
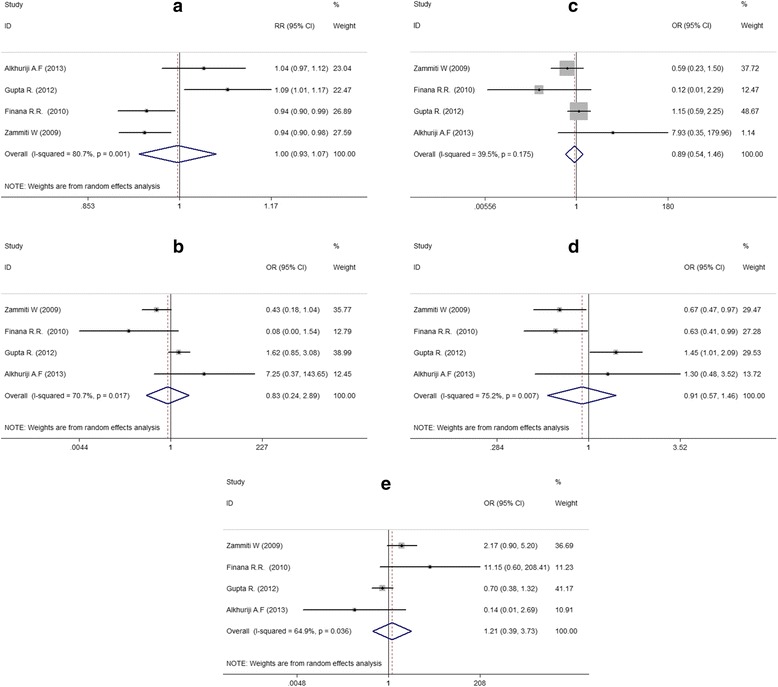
Forest plots for the associations between -238G/A polymorphism in the TNF-α gene and RPL risk (**a** codominant genetic models; **b** homozygous genetic models; **c** heterozygous genetic models; **d** dominant genetic models; **e** recessive genetic models)

In order to explore the potential correlation between genotypes of -238G/A and RPL, we compared data using all the genetic models (Fig. 3). Nevertheless, no significant association was observed. The results were as follows: A vs. G (OR = 0.996, 95% CI 0.932-1.065, *P* = 0.912), AA *vs.* GG (OR = 0.830, 95% CI 0.238-2.887, *P* = 0.769), AA *vs.* GA (OR = 0.888, 95% CI 0.54-1.46, *P* = 0.640), AA + GA *vs.* GG (OR = 0.908, 95% CI 0.566-1.456, *P* = 0.690), and AA *vs.* GA + GG (OR = 1.212, 95% CI 0.394-3.729, *P* = 0.737) (Table 2).

### Sensitivity analysis

**Fig. 4:**
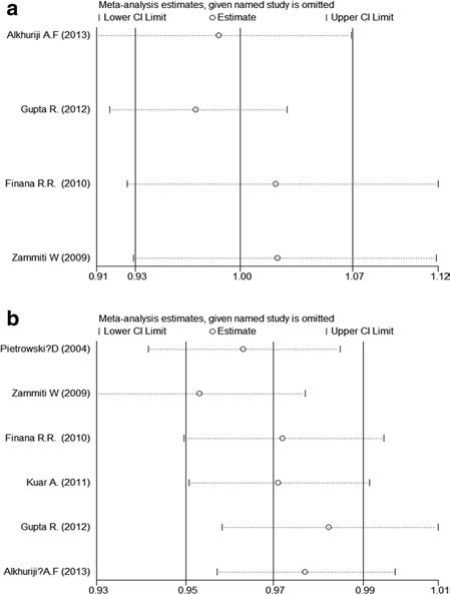
Sensitivity analysis for the associations between polymorphisms in the TNF-α gene and RPL risk (**a** -238G/A; **b** −308 G/A)

Sensitivity analysis was carried out to evaluate the stability of the overall results through sequential omission of individual studies. The results of sensitive analysis indicated that every single study did not influence the overall results qualitatively, demonstrating reliability and robustness of our results (Fig. 4).

### Publication bias evaluation

**Fig. 5:**
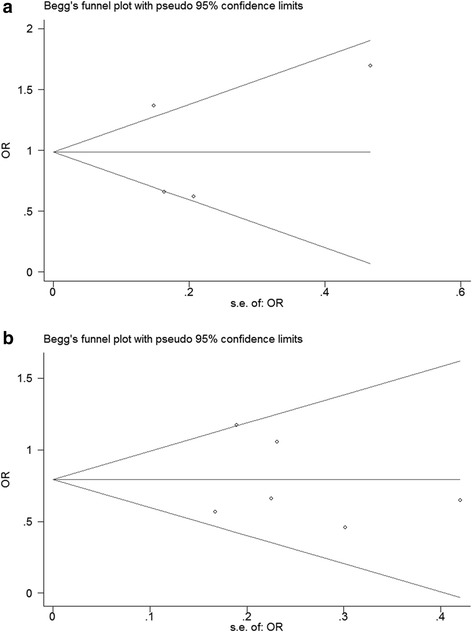
Funnel plot for the associations between polymorphisms in the TNF-α gene and RPL risk (**a** -238G/A, **b** −308 G/A)

Funnel plots and the Egger's test were employed to assess the publication bias of included studies. In the funnel plot analysis, the shape of funnel plot seemed symmetrical (Fig. 5). Furthermore, Egger's test did not display statistically significant publication bias for -238G/A (*t* = 0.18, *P* = 0.87) and −308 G/A (*t* = −0.33, *P* = 0.76).

## Discussion

RPL is a common disorder and represents a major concern for reproductive problem [38] affecting 1% to 3% of healthy women and occurs in 10% to 20% of pregnant woman [39]. Until now, various factors have been identified that influence miscarriage, however, the exact underlying etiology in up to 50% of RPL patients remains undetermined [38].

As a pleiotropic cytokine, TNF-α has attracted attention because of its involvement in the promotion of inflammatory response, autoimmune, endocrine and neoplastic diseases. Furthermore, there is convincing evidence that TNF-α induces apoptosis of cytotrophoblasts, which suggested that aberrant expression of TNF-α may have harmful effects on placental development and function [40]. Increasing evidence shows that TNF-α mediate a number of pregnancy complications including RPL [28, 41]. Mechanistically, increased TNF-α secretion led to RPL through inducing trophoblast invasion and placentation [42] and proapoptotic gene expression in human fetal membranes [43], resulting in accelerated membrane degradation and increased infertile susceptibility [44]. Moreover, regulated TNF-α expression in the developing placenta may interfere with pregnancy survival. Increased placental levels of TNF-α increases abortion rates [45], and blockade of TNF-α has been shown to prevent stress-induced miscarriage in mice [46]. Based on the above, TNF-α may be involved in the pathogenesis of RPL.

The incidence of RPL is controlled by genetic factors, and genetic polymorphisms have been associated with poor pregnancy outcome [16]. In the TNF-α gene, several polymorphisms had been identified, which might have a role in the pathogenesis of RPL, however, recent evidence dealing with the association of RPL and TNF-α gene polymorphisms presented some contradictory results. Several studies have reevaluated the connection between RPL risk and TNF-α polymorphisms [20, 34]. A recent meta-analysis by Alkhurijiet al. [17] suggested that the TNF-α gene polymorphism at position -308G/A could be a genetic predisposing factor for unexplained RPL while they found no association between TNF-α-238G/A polymorphism and RRL. In addition, several studies failed to find the association between the common polymorphisms in the TNF-α gene and RPL risk [19, 33]. Since the discrepant study designs and statistical methods, and the diversities in sample sizes, countries of origin might lead to unreliable results, this present meta-analysis aimed to provide a more comprehensive and reliable conclusion between TNF-α gene functional polymorphisms and RPL.

The present meta-analysis included 1430 cases and 1727 controls from 10 independent case–control studies. The results suggested that -308G/A polymorphisms related with an elevated risk of RPL, indicating that -308G/A may be risk factors for RPL. However, no statistically significant association was observed between -238G/A and RPL risk. One possible reason behind this pattern of results could be that -308G/A polymorphism were more impactful than -238G/A on TNF-α gene expression and protein production, thereby possibly contributing to RPL risk. Moreover, stratification by geographic position, the polymorphism of -308G/A was significantly associated with RPL risk for Asians rather than non-Asians. The reason for the discrepancy is unclear, but it might be explained in part by geographic variation in the frequency of the allele A, i.e., TNF-α-308G/A polymorphism in the Asian patients was higher than non-Asians (14.86 vs.9.63%). The difference could also be explained by the small sample sizes of several included studies, which may lead to substantial errors from estimation [22]. Thus, TNF-α-308G/A polymorphism may contribute to RPL susceptibility, especially in Asian population by the subgroup analysis.

In addition, no correlation was observed between -238G/A polymorphism and susceptibility to RPL. Smaller studies are often characterized by larger effects in this meta-analyses of -238G/A polymorphism, which can be possibly explained by publication bias [47]. It is possible that publication bias may pose a problem for meta-analyses [48]. Thus, more convincing evidences are required to draw a solid conclusion on the correlation between -238G/A polymorphism and the risk of RPL.

Our meta-analysis of the relationship between the TNF-α polymorphisms and RPL risk differs from the results previously reported by Zhang [21]. The previous data demonstrated that TNF-α-308G/A, −238G/A polymorphisms are not associate with the risk of RPL in the overall population. This may be because the present study included five more studies and removed studies where diagnostic criteria of RPL were at least two consecutive spontaneous abortions [33–37]. Our meta-analysis revealed, however, that the TNF-α-308G/A polymorphism is associated with susceptibility to RPL, especially in Asian populations, suggesting that TNF-α may play a role in RPL susceptibility.

As with other meta-analyses, it was prudent to acknowledge that several potential limitations were apparent in this analysis. First, the number of studies and the sample sizes were relatively small for analysis of each gene polymorphism thereby having insufficient power to estimate the association between TNF-α genetic polymorphisms and RPL risk. Second, a meta-analysis is a retrospective study, the selection bias would lead to the heterogeneity of the results, and thereby possibly influencing the reliability of our conclusions. Even though the studies have similar inclusion criteria, there are also some differences such as different examinations of each group patients, potential confounders (i.e., age, race) might skew the results. Finally, our study only included articles published in English from the three selected databases, which might limit the results of the meta-analysis. It was critical that larger and well-designed studies should be performed to reevaluate the association precisely.

## Conclusion

In summary, this meta-analysis systematically evaluated the association between TNF-α genetic polymorphisms and RPL risk and demonstrates that -308G/A polymorphism in the TNF-α gene is associated with susceptibility to RPL. This polymorphism might be a risk factor for RPL. Further functional studies between TNF-α gene and RPL risk are warranted.

## Abbreviations

TNF-α: tumor necrosis factor alpha
RPL: recurrent pregnancy loss
OR: odds ratio
CI: Confidence intervals
SNP: single nucleotide polymorphisms
SEM: Standard error of the mean
vs.: Versus
PCR-RELP: polymerase chain reaction-restriction fragment length polymorphism
